# Systems biology approach unveils the cellular and molecular mechanisms of formalin-inactivated whole cell vaccine-induced protective immunity against *Coxiella burnetii* infection in mice

**DOI:** 10.3389/fimmu.2026.1809423

**Published:** 2026-06-04

**Authors:** Venkatesh Kumaresan, Duolin Wang, Yan Zhang, Dong Xu, Guoquan Zhang

**Affiliations:** 1 Department of Molecular Microbiology and Immunology, University of Texas at San Antonio, San Antonio, TX, United States; 2 Department of Electrical Engineering and Computer Science, University of Missouri-Columbia, Columbia, MO, United States; 3 Informatics Institution, Morsani College of Medicine, University of South Florida, Tampa, FL, United States; 4 Bellini College of Artificial Intelligence, Cybersecurity and Computing, Morsani College of Medicine, University of South Florida, Tampa, FL, United States

**Keywords:** balb/c mice, Coxiella burnetii, formalin-inactivated vaccine, neutrophil, Nine Mile phase II, Nine Mile phase I, RNA-seq

## Abstract

**Background:**

The formalin-inactivated *Coxiella burnetii* virulent phase I vaccine (PIV) has been shown to be more protective than the avirulent phase II vaccine (PIIV) against virulent *C. burnetii* challenge in animal models. However, the cellular and molecular mechanisms underlying the differential ability of PIV and PIIV to induce protective immunity remain unclear.

**Methods:**

PIV and PIIV were generated from axenic (ACCM-D) cultures and administered to mice using single or multiple immunization regimens. Protective efficacy was evaluated following challenge with virulent *C. burnetii*. Humoral immune responses were assessed by measuring phase I–specific IgM and IgG antibodies. Bulk RNA sequencing and flow cytometry were used to compare immune responses in PIV- and PIIV-vaccinated mice. The role of neutrophils in vaccine-mediated protection was examined through neutrophil depletion prior to challenge.

**Results:**

Regardless of single or multiple immunizations, PIV and PIIV generated from axenic (ACCM-D) cultures retained their differential protective efficacies, with PIV providing robust protection but PIIV did not. PIV elicited earlier phase I-specific IgM responses and sustained higher IgG responses compared to PIIV, indicating differences in immunogenicity. Cellular and transcriptomic analyses revealed that PIV induced a prolonged neutrophil response in the spleen, accompanied by upregulation of genes involved in neutrophil degranulation, metal sequestration, and TLR-dependent innate immune pathways. In contrast, the neutrophil response induced by PIIV was transient and did not persist into later stages of vaccination. Depletion of neutrophils in PIV-vaccinated mice prior to challenge significantly reduced the protective efficacy of PIV.

**Conclusion:**

These findings demonstrate that PIV-induced protection against *C. burnetii* infection is partially dependent on sustained neutrophil activation. This study provides novel evidence that modulation of neutrophil-mediated effector functions plays a critical role in PIV-mediated protective immunity.

## Introduction

*Coxiella burnetii* is an obligate intracellular bacterium that causes the worldwide zoonotic disease, Q fever. The abilities of *C. burnetii* to spread through aerosols with extremely low infectious dose and resistance to environmental conditions make this pathogen a potential biothreat agent (1). Therefore, *C. burnetii* is classified as a Tier 2 Select Agent by the Centers for Disease Control and Prevention and the Department of Health and Human Services in the US. Clinically, Q fever commonly presents as an acute flu-like illness but it can progress into chronic diseases, such as atypical pneumonia, hepatitis, endocarditis and encephalitis, particularly in immuno-compromised individuals (2). Acute Q fever is treatable with doxycycline for two to three weeks, whereas chronic Q fever requires prolonged combination antibiotic therapy, often lasting at least for 18 months (3). Even after successful antibiotic treatment, the risk of recurrent *C. burnetii* infection remains high (4). Most human infections arise through inhalation of aerosols generated from contaminated dust or mist from animal urine, feces, birth fluids, or placentas (especially from sheep and goats). Thus, handling contaminated materials like straw, wool, or fertilizer, or having direct contact with infected animals during birth poses a high risk for *C. burnetii* infection. Additionally, human infection can occur through consumption of unpasteurized milk from infected animals and through tick bites (5).

To date, although over 70 genotypes of *C. burnetii* strains have been identified, the Nine Mile strains have been the most extensively studied strains (6). The Nine Mile strains exist as two phase variants distinguished by their lipopolysaccharide (LPS) structures: the virulent Nine Mile phase I (NMI) variant expresses full-length LPS, whereas the avirulent phase II (NMII) variant contains truncated LPS (7, 8). Genomic analyses show that NMII lacks homologs of genes CBU_0679 to CBU_0697, which are present in NMI and are largely involved in LPS biosynthesis (9). Consistent with these genomic differences, NMI induces fever and inflammation in immunocompetent mice and guinea pigs, whereas NMII does not (10, 11).

Q-VAX^®^, a formalin-inactivated whole-cell vaccine produced from the Henzerling Phase I strain is the only licensed vaccine for humans that can provide near-complete protection against *C. burnetii* natural infections (12). However, Q-VAX frequently causes adverse reactions, including subcutaneous swelling, erythema, and induration at the site of inoculation, particularly when administered to individuals with pre-existing immunity to *C. burnetii* (13). Consequently, all potential vaccinees are required to undergo screening for pre-existing immunity by the Q-VAX^®^ Skin Test prior to immunization. In veterinary settings, Chlamyvax FQ^®^, an oil-emulsion vaccine containing *Chlamydophila abortus* and inactivated phase II *C. burnetii*, is licensed for animals in France (14). However, Chlamyvax FQ has shown limited protective efficacy, as it failed to prevent abortion or reduce *C. burnetii* shedding in milk, feces, placenta, or vaginal secretions (15). Despite the fact that the formalin-killed phase I whole-cell vaccine induces stronger protective immunity, the cellular and molecular mechanisms underlying its ability to induce protective immunity remain unclear.

The immune system is a complex, multilayered network comprising biochemical signaling pathways and coordinated interactions among cells, tissues, and organs (16). The spleen plays a central role in protective immunity, comprising diverse myeloid populations, including dendritic cells, neutrophils, eosinophils, monocytes, and macrophages, as well as lymphoid populations such as B and T cells (17). In addition, immune cell phenotyping using flow cytometry has provided a robust method to monitor immune responses within mixed cell populations and to define phenotypic and functional characteristics of individual cell subsets using fluorochrome-conjugated antibodies targeting specific antigens (18). By profiling myeloid and lymphoid markers, flow cytometry enables detailed characterization of vaccine-induced immune responses in the spleen. Complementarily, bulk RNA-seq offers quantitative insight into intracellular transcriptional networks in the spleen, allowing comparative analysis of responses to vaccines, and adjuvant controls (19). Integrating these approaches has the potential to identify the cellular and molecular networks underlying vaccine-induced protective immunity against *C. burnetii* as well as to discover uncover new targets for vaccine development against Q fever.

Our previous study demonstrated that the formalin-inactivated *C. burnetii* Nine Mile phase I (PIV) and phase II (PIIV) vaccines derived from an acidified citrate cysteine medium (ACCM-2) culture retained their protective efficacies (20). In this study, we demonstrated that regardless of single or multiple immunization, PIV and PIIV derived from axenic (ACCM-D) culture also retained their different protective efficacies. To identify the cellular and molecular differences responsible for the distinct protection induced by PIV but not by PIIV, systems biology approaches were used to compare the immune responses between PIV- and PIIV-vaccinated mice. Bulk RNA-seq and flow cytometry analyses indicated that, compared to the non-protective PIIV, protective PIV elicited prolonged recruitment and activation of neutrophils in the mouse spleen, which was accompanied by upregulation of genes involved in neutrophil degranulation, metal sequestration, and TLR-dependent innate immune pathways. The observation that depletion of neutrophils in PIV-vaccinated mice before challenge with virulent *C. burnetii* significantly reduced the ability of PIV to provide protection in mice suggests that neutrophils contribute to vaccine-induced protective immunity against Q fever. Thus, this study provides novel evidence supporting that the ability of PIV to modulate neutrophil function may play an important role in PIV-induced protective immunity against *C. burnetii* infection.

## Materials and methods

### Animal

Eight-week-old BALB/c mice were purchased from the Jackson Laboratory (Bar Harbor, ME). Mice were housed in sterile microisolator cages with five mice per cage under pathogen-free conditions at the University of Texas at San Antonio BSL-3 laboratory animal facility and fed according to University regulations. All procedures were approved by the UTSA Institutional Biosafety Committee and the Animal Care and Use Committee.

### Vaccination

Whole cell vaccines of NMI (PIV) and NMII (PIIV) were prepared by inactivating purified *C. burnetii* NMI and NMII with 1% formaldehyde, as described previously (21). Mice were divided into four groups (PBS, Adjuvant, PIV, PIIV), each with four biological replicates. Vaccination was administered subcutaneously: mice were restrained by scruff, and 10 µg of PIV or PIIV in 50 µL PBS plus 50 µL aluminum hydroxide gel (Alum) adjuvant (Invivogen) was injected into the scruff skin to a depth of 0.5 cm using a sterile 25G needle. The adjuvant group received 50 µL PBS with 50 µL adjuvant, and the PBS group received 100 µL PBS.

### C. burnetii challenge and necropsy

Virulent NMI bacteria grown in ACCM-D were used for mouse challenges. Mice were anesthetized by using isoflurane delivered in oxygen and intraperitoneally injected with 1×10^7^ genome equivalents (GE) of NMI in 400 µL PBS. Body weight was recorded at the time of injection (Day 0) followed by 3-, 7-, 10-, and 14-days post-infection. After 14 days post infection (dpi), mice were euthanized by CO_2_ exposure. Spleens were dissected, weighed, and splenomegaly were determined using the following formula: (% of spleen weight/body weight). Forty mg of spleen tissue was used for genomic DNA quantification. Blood was collected by cardiac puncture, and serum was separated by centrifugation at 1,500 × g for 10 minutes at room temperature. Spleens and serum were stored at –20 °C if not processed immediately.

### Genomic DNA copy number analysis by qPCR

Spleen samples were homogenized in 200 μL of lysis buffer (1 M Tris, 0.5 M EDTA, 7 mg/mL glucose, 28 mg/mL lysozyme) and filtered through a 100-μm nylon mesh to remove connective tissue. Ten microliters of proteinase K (20 mg/mL) were added, and samples were incubated at 60 °C for 18 hours. Twenty-one microliters of 10% SDS were then added, followed by incubation at room temperature for 1 hour. DNA was subsequently extracted using the High Pure PCR Template Preparation Kit (Roche, Indianapolis, IN) according to the manufacturer’s instructions.

Bacterial burden was quantified using a TaqMan assay targeting the *C. burnetii* *com1* (CBU_0680) gene, normalized to the mouse tfrc gene. Custom TaqMan™ RNA Assays (FAM-labeled for *CBU_0680*) were obtained from Invitrogen, and the TaqMan™ Copy Number Reference Assay (VIC-labeled mouse tfrc) was used for normalization. qPCR was performed on an Applied Biosystems QuantStudio 3 real-time PCR system. Standard curves were generated using recombinant plasmids containing *CBU_0680*, or tfrc sequences cloned into the pBluescript vector. Results are expressed as log_10_ gene copy number (10). All experiments were performed in triplicate, including both technical and biological replicates.

Statistical analyses were performed using R. Differences among groups (Adjuvant, PIV, and PIIV) were assessed using one-way analysis of variance (ANOVA) followed by *post hoc* pairwise comparisons. When a significant overall effect was detected, pairwise differences between groups were evaluated using Tukey-adjusted comparisons based on estimated marginal means. Results are presented as individual data points overlaid on boxplots, showing the median, interquartile range, and distribution of values for each group. Statistical significance was defined as p < 0.05. Graphical visualization and statistical annotations were generated using ggplot2 and ggpubr.

### ELISA

Serum samples were collected at 0, 3-, 7-, 14-, 21-, and 28-days post-vaccination (dpv) and analyzed for IgM, IgG, IgG1, IgG2a, and IgG3 against PI antigens by ELISA. Briefly, 50 µL of inactivated PI (500 ng/mL in 0.05 M carbonate/bicarbonate buffer, pH 9.6) was coated on 96-well plates at 4 °C for 48 h. Plates were blocked with 1% BSA in PBST (0.05% Tween-20 in PBS) and incubated with 100 µL of serially diluted mouse serum at room temperature for 2 h. After four washes with PBST, 100 µL of HRP-conjugated goat anti-mouse IgM, IgG, IgG1, IgG2a, or IgG3 (1:5,000 dilution) was added for 2 h at room temperature. Substrate was Sigma Fast O-Phenylenediamine Dihydrochloride (Sigma-Aldrich), and optical density was measured at 490 nm using the SpectraMax M2 system (Molecular Devices). Statistical analyses were performed using R. Antibody responses were analyzed using two-way analysis of variance (ANOVA) with Group and Time as factors, including their interaction. When appropriate, *post hoc* comparisons between vaccinated groups (PIV and PIIV) and the Adjuvant control were performed using estimated marginal means with Tukey-adjusted pairwise tests within each time point. Data are presented as mean ± standard error of the mean (SEM). Differences were considered statistically significant at p < 0.05. Graphical representations were generated using ggplot2.

### Splenocyte isolation

After 7 and 28 dpv, mice were euthanized by CO_2_ exposure followed by cervical dislocation. Spleens were homogenized through a 70 µm cell strainer, red blood cells were lysed using ACK buffer, and cells were centrifuged at 400 × g for 10 minutes. Viable cells were counted using a hemocytometer and Trypan blue, with >98% viability for all assays.

### RNA isolation and Illumina sequencing

Immediately after cell separation, 5x10^6^ cells of splenocytes from each group (N = 5) were pooled together and total RNA was isolated using the RNeasy Mini Kit (Qiagen). Contaminating DNA was removed with the TURBO DNA-free Kit (Ambion), and RNA samples were processed for Illumina sequencing.

### RNA-seq

The integrity of isolated total RNA was assessed by capillary electrophoresis fragment analysis (University of Missouri DNA Core Laboratory), and both the 28S/18S rRNA ratio and RNA integrity number (RIN) were calculated. All samples exhibited RIN scores above 9.5 and were subsequently used for library preparation. Strand-specific RNA-seq libraries were generated using the Illumina TruSeq HT Stranded Total RNA Library Prep Kit (Illumina, San Diego, CA) according to the manufacturer’s instructions. Libraries were normalized, pooled, and sequenced on an Illumina HiSeq 2000 using a 100-nucleotide paired-end protocol.

Raw reads were aligned to the mouse reference genome (GRCm38, Ensembl) using STAR, and sorted with SAMtools. Strand-specificity was verified using RSeQC v2.6.4 in conjunction with the mouse RSeQC reference file (GRCm38_mm10_Ensembl.bed.gz). Raw gene-level counts were normalized using HTSeq with 0.75 quartile normalization, log_2_-transformed, and are available in the Gene Expression Omnibus (GEO) under accession number GSE315405. Differential gene expression (DEG) between groups was calculated using GFold, and genes exhibiting ≥3-fold change were considered significant (21).

Pathway enrichment and protein network analyses for DEGs were performed using STRING v11 and KEGG databases (22, 23). Multi-dimensional scaling (MDS) plots and heatmaps were generated in R using the limma and pheatmap packages to visualize sample relationships and expression patterns. All RNA-seq statistical analyses were conducted in R (v4.2) unless otherwise specified.

### Flow cytometry

Splenocyte populations (T cells, B cells, monocytes, granulocytes, dendritic cells) were analyzed by flow cytometry. Cells were adjusted to 1×10^6^ per 200 µL in 96-well round-bottom plates, centrifuged at 500 × g for 10 min, and Fc-blocked for 15 min. Cells were stained for 30 min with the following antibody panels: Panel 1: anti-CD3-PE-Cy7, anti-CD8-APC-EF780, anti-CD4-FITC, anti-CD25-PE, anti-CD44-APC; Panel 2: anti-CD19-AF647, anti-CD9-PE, anti-CD27-AF488, anti-MHCII-PE-cy7; Panel 3: anti-CD11b-PE-vio770, anti-CD14-FITC, anti-Ly6G-APC-Cy7, anti-siglecF-Alexa Fluor 647, anti-F4/80-PE-Cy5, anti-CD125-PE and Panel 4: anti-CD11c-PE-EF610, anti-CD8-FITC, anti-CD68-APC, anti-MHCII-PE-Cy7. After staining for 30 minutes, the cells were washed twice with MACS buffer (Miltenyi Biotech, Germany) and the labelled cells were analyzed on CyAn (Beckman Coulter Inc, USA) or BD Fortessa (BD Biosciences, USA) using FACSDiva acquisition software (BD Biosciences, USA). Data analyses were performed using Summit 5.2 (Beckman Coulter Inc, USA) and FlowJo v10.10.1 (Tree Star). Statistical analyses were performed using R. Cellular levels were analyzed using two-way ANOVA with Group (Control, Adjuvant, PIV, PIIV) and Time (7 and 28 dpv) as fixed factors, including their interaction term. Analyses were conducted separately for each marker. When significant main effects or interactions were observed, *post hoc* pairwise comparisons between groups within each time point were performed using estimated marginal means (EMMs) with appropriate multiple-comparison adjustment. Results are presented as mean ± standard error where applicable. Statistical significance was defined as p < 0.05. Data visualization was performed using bar graphs stratified by group and time. All analyses were conducted in R using the aov, emmeans, dplyr, and ggplot2 packages.

### Neutrophil depletion

Neutrophils were depleted using an anti-mouse Ly6G (1A8) monoclonal antibody (Leinco Technologies) and rat IgG2a were used as the isotype control. Eight-week-old BALB/c mice (N = 5 per group) were vaccinated with PIV or adjuvant alone for 28 days. Following the vaccination period, mice were challenged intraperitoneally with 1×10^7^ GE of NMI dissolved in 400 µL PBS. One day before the NMI challenge, each mouse received an intraperitoneal injection of 200 µg Ly6G or IgG2a antibody diluted in 200 µL buffer (pH 6.5) (Bio X Cell, NH, USA). Antibody administration continued every other day until necropsy (24). Body weight was recorded on days 0, 3, 7, 10, and 14 post-challenge. At 14 days post-challenge, mice were euthanized by CO_2_ exposure, the spleens were collected and weighed, and 40 mg of spleen tissue was used for genomic quantification. Statistical analysis was performed using One-way ANOVA as mentioned in the earlier section.

## Results

### Repeated vaccination reveals robust protection by PIV, but not PIIV, against virulent NMI

**Figure 1:**
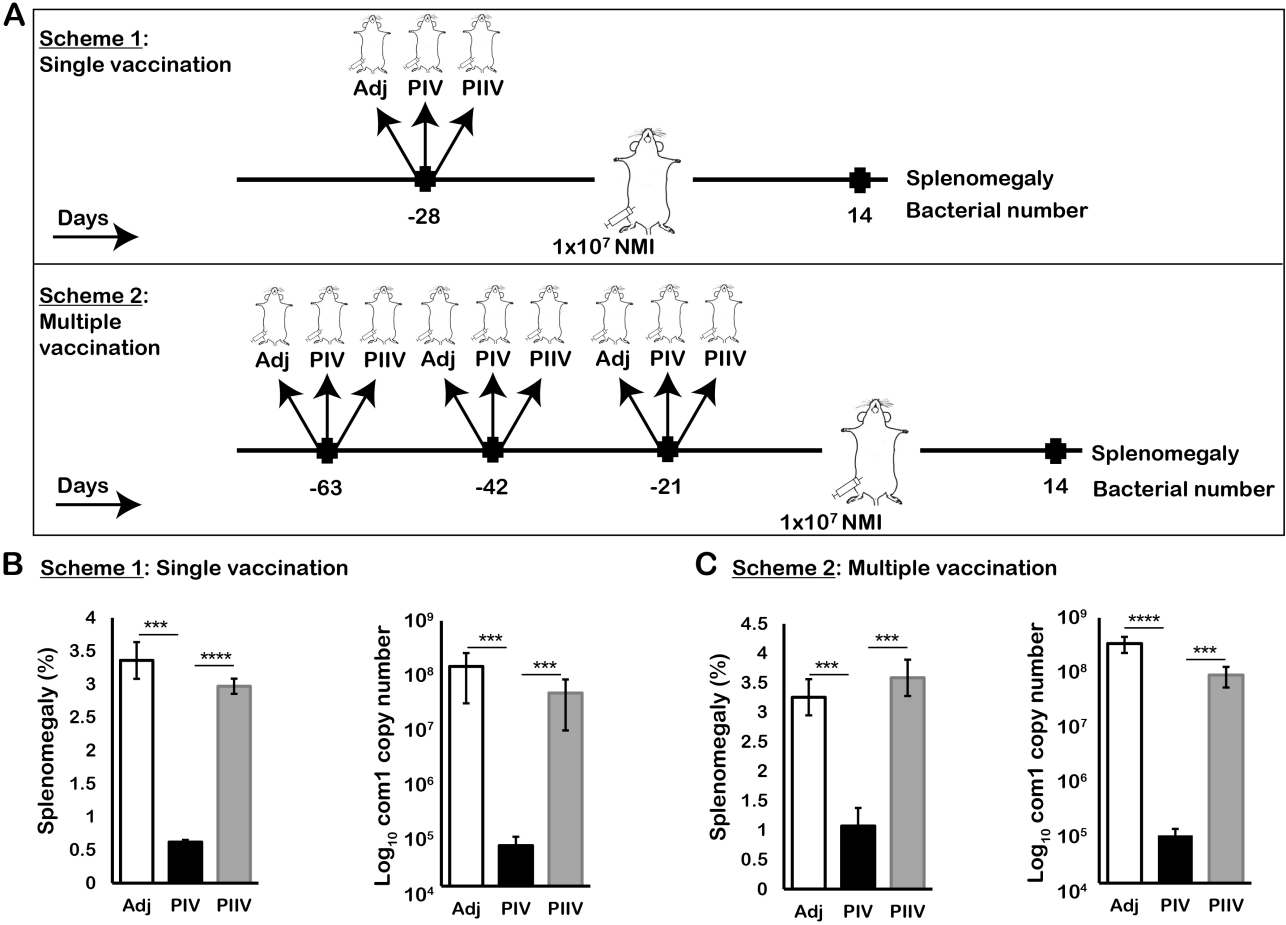
Single and multiple vaccinations of PIV but not PIIV reduced splenomegaly and bacterial burden in mice. **(A)** Vaccination schemes used for single and multiple vaccinations. **(B, C)** Splenomegaly (spleen weight/body weight, %) and bacterial burden (relative genomic copy number of *CbCom1* normalized to mouse *tfrc*, determined by qPCR) measured at 14 dpi in mice receiving **(B)** single vaccination or **(C)** multiple vaccinations. Data are presented as mean ± SEM (N = 5 per group). Statistical significance was determined using one-way analysis of variance (ANOVA) followed by *post hoc* pairwise comparisons; ****p < 0.0001 and ***p < 0.001.

Our previous studies (8, 21) demonstrated that PIV confers significant protection against virulent *C. burnetii* challenge, whereas PIIV does not. However, the comparative efficacy of single versus repeated vaccinations with PIV and PIIV against NMI infection in mice has not been examined. To address this, eight-week-old BALB/c mice (N = 5) were immunized subcutaneously with 10 µg PIV or 10 µg PIIV formulated with Alum, or with Alum alone. Two vaccination schemes were tested to evaluate single- and booster-dose efficacy (**Figure 1A**): mice were challenged intraperitoneally with 1 × 10^7^ NMI either 28 days after a single vaccination or 21 days after two booster doses administered at 21-day intervals. Fourteen days post-challenge, splenomegaly and bacterial burden were assessed.

In both vaccination schemes, PIV-vaccinated mice exhibited significantly reduced splenomegaly and lower bacterial loads compared with Alum-immunized controls (**Figures 1B, C**). In contrast, PIIV-vaccinated mice displayed splenomegaly and bacterial burdens comparable to Alum controls, indicating that, regardless of single or repeated immunization, PIIV did not confer protection against virulent *C. burnetii* NMI challenge. These results confirm that PIV and PIIV differ in their capacity to induce protective immunity against virulent *C. burnetii* infection.

### PIV immunized mice produce robust PI-specific IgG antibodies than PIIV

**Figure 2:**
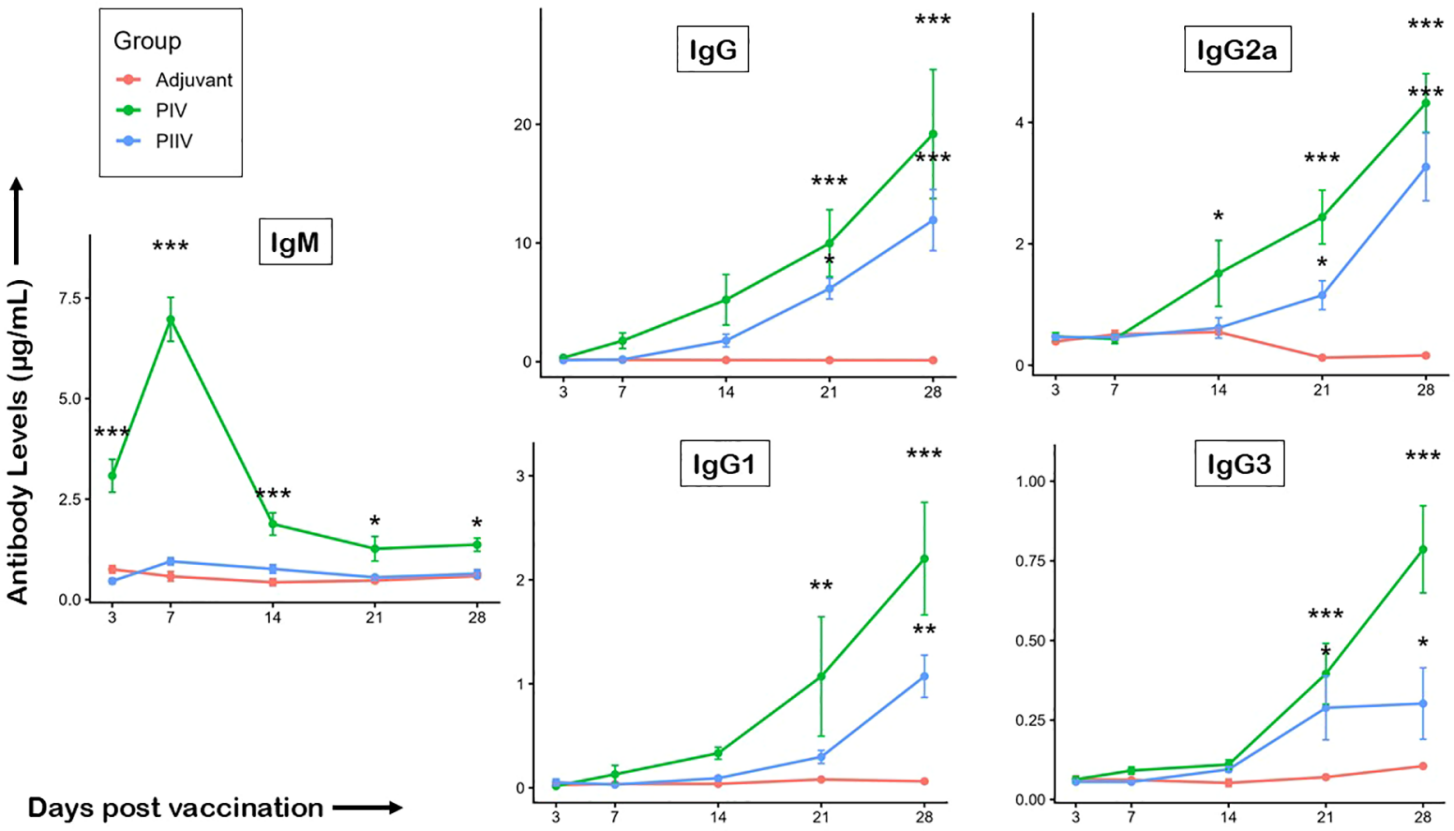
PIV induces robust IgM, IgG, and IgG subtype antibody responses. Serum concentrations of *C. burnetii* NMI-specific IgM **(A)**, IgG **(B)**, IgG1 **(C)**, IgG2a **(D)**, and IgG3 **(E)** were measured in mice vaccinated with a single dose of PIV or PIIV, compared to adjuvant-only controls. Data are presented as mean ± SEM (N = 5 per group). Statistical significance was determined using two-way ANOVA; ***p < 0.001, **p < 0.01 and *p < 0.05.

To determine whether PIV and PIIV elicit distinct antibody responses, eight-week-old BALB/c mice (N = 5) were vaccinated subcutaneously with 10 µg PIV or 10 µg PIIV formulated with Alum, or with Alum alone. Serum samples were collected at 3, 7, 14, 21, and 28 dpv and analyzed for PI-specific IgM, IgG, and IgG subclasses (IgG1, IgG2a, and IgG3) by ELISA. As shown in **Figures 2A, B**, PIV-vaccinated mice generated significantly higher levels of PI-specific IgM and IgG antibodies than PIIV-vaccinated mice at early time points (3 and 7 dpv). By 14 and 28 dpv, IgM levels declined in both groups, whereas IgG and its subclasses continued to increase over time. Among the IgG subclasses, IgG2a was the most abundant, followed by IgG1, with IgG3 being the least produced in both PIV- and PIIV-vaccinated mice (**Figures 2C–E**). These findings indicate that PIV induces more rapid and sustained PI-specific antibody responses than PIIV. The predominance of IgG1 and IgG2a subclasses suggests that these isotypes may contribute to PIV-mediated protective immunity.

### PIV elicited a stronger prolonged inflammatory response than PIIV in mice

**Figure 3:**
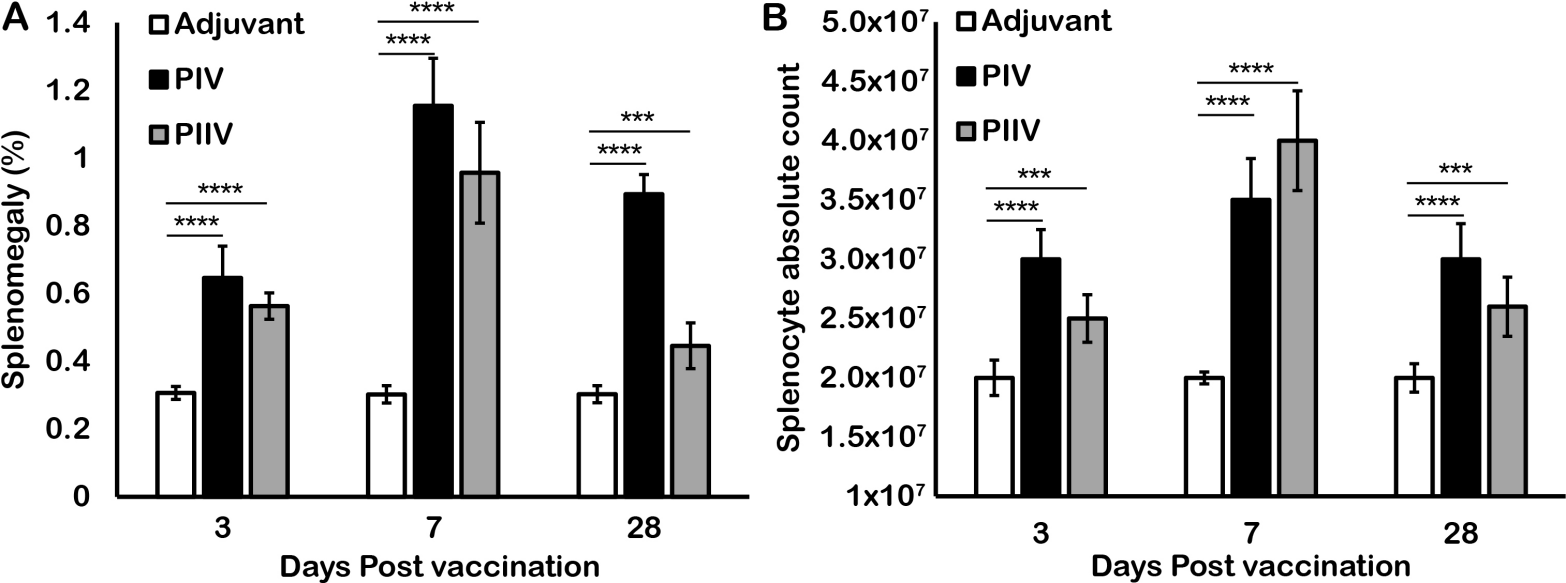
PIV induces higher splenic response than PIIV. Graphs showing the **(A)** Splenomegaly (%) and **(B)** Splenocyte counts quantified from PIV and PIIV vaccinated mice at 3, 7 and 28 dpv compared to adjuvant. Statistical significance was determined using two-way ANOVA; ****p < 0.0001 and ***p < 0.001.

To investigate the cellular basis of PIV-induced protection, eight-week-old BALB/c mice (N = 5) were vaccinated subcutaneously with 10 µg PIV or 10 µg PIIV formulated with Alum, or with Alum alone. Splenomegaly was assessed at 3, 7, and 28 dpv. As shown in **Figure 3A**, both PIV- and PIIV-vaccinated mice displayed increased spleen size compared with Alum controls at all time points. By 28 dpv, however, splenomegaly remained elevated in PIV-vaccinated mice, whereas it had decreased significantly in PIIV-vaccinated mice. Total splenocyte counts, assessed as an additional measure of immune activation (**Figure 3B**), were consistent with these observations. Both vaccine groups had higher cell numbers than Alum controls, with PIV-vaccinated mice maintaining the highest counts at 28 dpv. These results indicate that PIV vaccination induces a more sustained splenic immune response than PIIV, which may contribute to the observed differences in protective efficacy.

### PIV and PIIV differentially stimulated neutrophil response in mice

**Figure 4:**
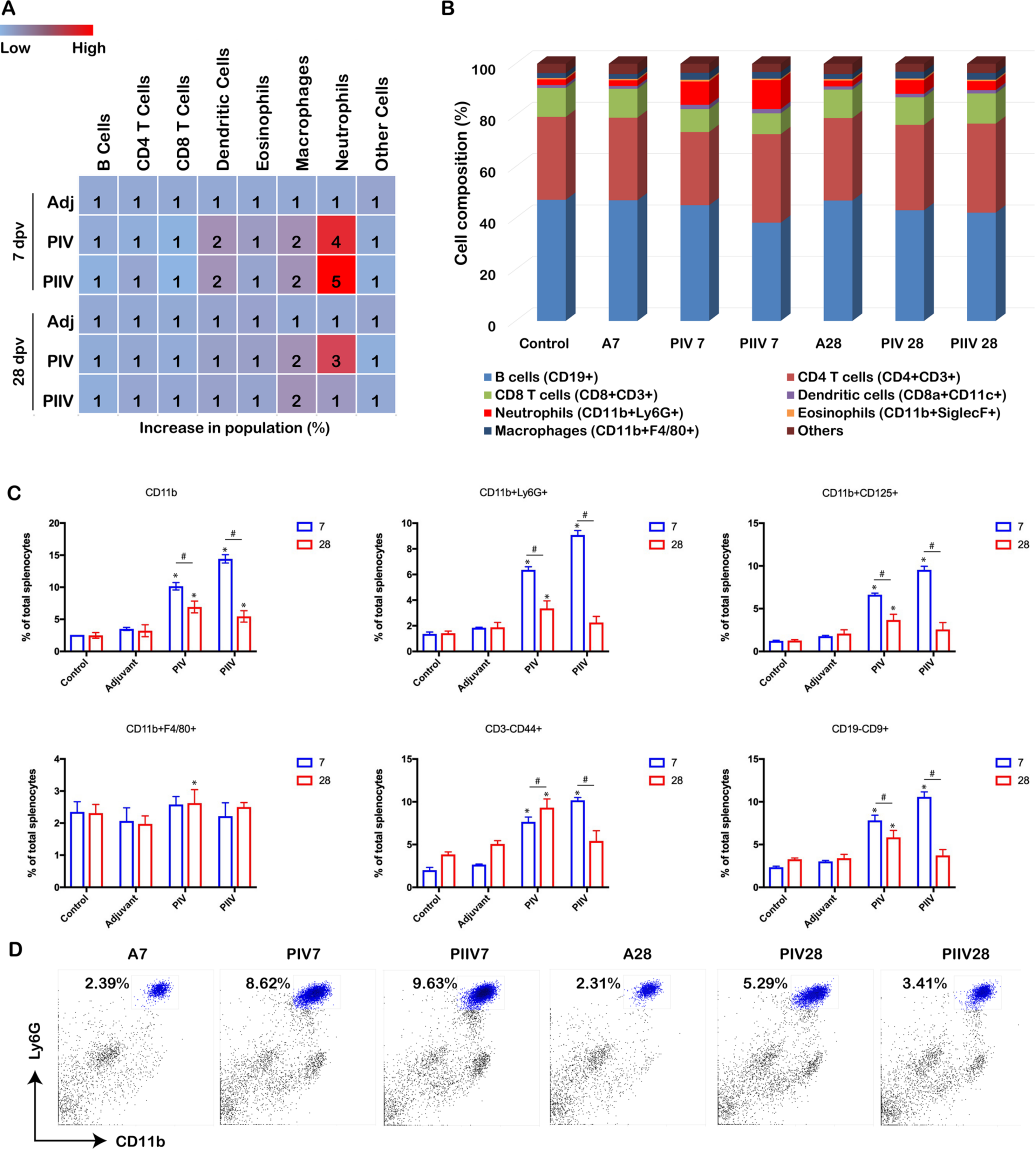
Immune cell composition in spleens of PIV- and PIIV-vaccinated mice. **(A)** Heatmap showing fold differences in splenic immune cell populations in mice vaccinated with PIV, PIIV, or adjuvant at 7- and 28-dpv relative to naïve mice, with red indicating high abundance and purple indicating low abundance, as determined by flow cytometry. **(B)** Bar graph showing the percentage of each immune cell type in spleens of PIV-, PIIV-, adjuvant-vaccinated, and naïve mice at 7 and 28 dpv. **(C)** Bar graphs showing the percentage of live cells for each cell type in spleens of PIV-, PIIV-, adjuvant-vaccinated, and naïve mice at 7 and 28 dpv. Statistical significance was assessed using two-way ANOVA with Group and Time as factors; * represent comparison with adjuvant and # represent comparison between 7 and 28 dpv. **(D)** Dot plots showing CD11b^+^Ly6G^+^ cell populations in splenocytes from mice vaccinated with adjuvant, PIV, and PIIV at 7 and 28 days post-vaccination (dpv). The percentage of total splenocytes is indicated adjacent to each gated population.

To comprehensively characterize vaccine-induced alterations in splenic immune responses, we performed flow cytometry on splenocytes from PIV- and PIIV-vaccinated mice (n = 4) at 7 and 28 days post-vaccination (dpv), compared to adjuvant and naïve control mice. Three panels of antibodies were used to quantify T cell, B cell, and myeloid compartments. Data were analyzed using complementary visualization approaches, including fold-change heatmaps, relative cellular composition, and absolute frequencies. As shown in **Figures 4A**, fold-change heatmap analysis revealed coordinated modulation of innate immune cell populations following vaccination. At 7 dpv, both PIV- and PIIV-vaccinated mice exhibited comparable increases in dendritic cells, macrophages, and neutrophils relative to alum-immunized controls. By 28 dpv, macrophage frequencies remained elevated in both vaccine groups; however, sustained neutrophil enrichment was observed only in PIV-vaccinated mice. In contrast, adaptive immune populations, including B cells, CD4^+^ T cells, and CD8^+^ T cells, showed minimal differences between PIV- and PIIV-vaccinated groups at either time point, indicating that vaccination primarily influenced the quantity of innate cells rather than adaptive immune compartments in spleen during this window.

To determine how these changes affected the overall splenic immune landscape, relative cell composition was assessed using stacked bar plots (**Figure 4B**). This analysis revealed a pronounced shift in immune cell composition in PIV-vaccinated mice at 28 dpv compared to both alum-immunized and PIIV-vaccinated groups, suggesting that PIV vaccination preferentially alters the balance of innate immune populations rather than uniformly expanding all leukocyte subsets (**Supplementary Figure 1**).

To evaluate whether these compositional changes reflected true increases in cell abundance, absolute frequencies were quantified among live splenocytes following appropriate gating strategies (**Supplementary Figures 2**–**4**). Among all the analyzed cell types, neutrophils (CD11b^+^Ly6G^+^) were significantly increased in both PIV- and PIIV-vaccinated mice at 7 dpv compared to adjuvant (**Figures 4C, H**); however, by 28 dpv, this increase persisted only in PIV-vaccinated mice. A similar trend was observed for CD11b^+^CD125^+^ cells, which were selectively enriched in PIV-vaccinated mice at the later time point (**Figure 4D**). Co-expression analysis within the CD11b^+^ gate revealed an increased proportion of Ly6G^+^CD125^+^ cells, suggesting that these cells largely represent neutrophils expressing CD125 (**Supplementary Figure 4**). However, CD11b^+^F4/80^+^ macrophages showed less significant changes at 7 dpv but no changes at 28 dpv, suggesting less prominent role for splenic macrophages in PIV-vaccinated immune response (**Figure 4E**). Consistent with sustained innate activation, PIV-vaccinated mice exhibited increased frequencies of CD3^-^CD44^+^ (**Figure 4F**) and CD19^-^CD9^+^ (**Figure 4G**) populations at 28 dpv compared to PIIV-vaccinated mice. These populations represent activated non-T and non-B cell compartments, respectively, enriched for innate and myeloid-associated cells. Together, these findings indicate that PIV vaccination promotes prolonged activation of innate immune populations at later time points.

Collectively, these flow cytometry analyses demonstrate that while both vaccines induce an early, transient activation of innate immune responses, PIV vaccination uniquely sustains an expansion of total splenocytes, accompanied by increased frequencies of neutrophils and related innate cell populations. This prolonged innate activation may contribute to the enhanced protective efficacy of PIV against *C. burnetii* infection in mice.

### PIV and PIIV differentially activated neutrophil-related genes in murine splenocytes

**Figure 5:**
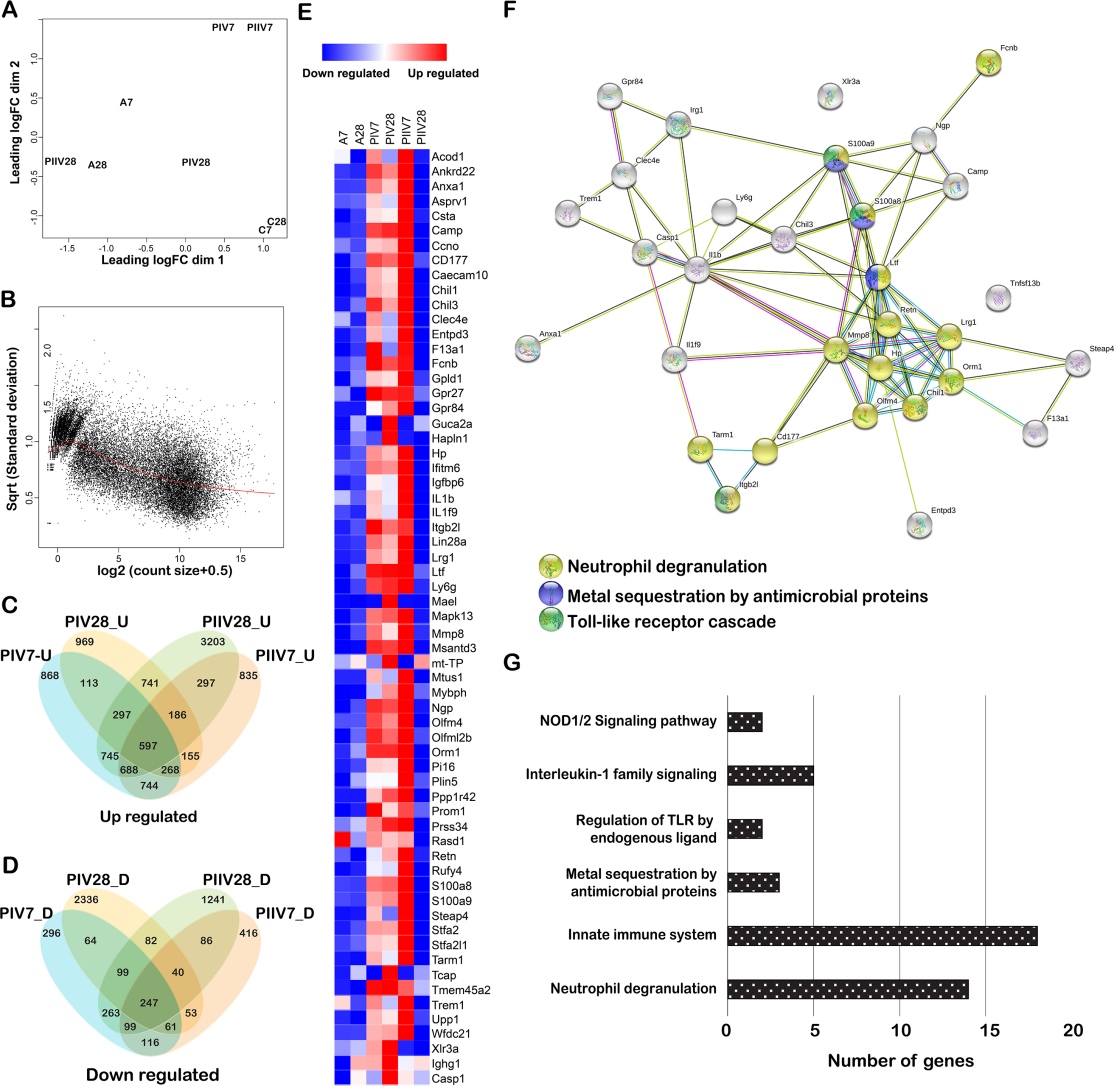
Transcriptomic profiling of splenocytes from PIV-, PIIV-, and adjuvant-vaccinated and naïve control mice. **(A)** MDS plot illustrating overall transcriptional similarity and separation among PIV, PIIV, adjuvant, and naïve control samples at 7 and 28 days post vaccination. **(B)** Mean-variance trend of log-transformed counts generated using the *voom* method, with gene-wise means and variances shown as black points and a LOWESS trend line overlaid. The broad dispersion of points reflects substantial biological variability across treatment groups. **(C, D)** Venn diagrams showing the number of uniquely upregulated **(C)** and downregulated **(D)** genes in splenocytes from PIV- and PIIV-vaccinated mice at 7 and 28 dpv. **(E)** Heatmaps highlighting differential expression of top DEGs in PIV-vaccinated splenocytes at 7 and 28 dpv relative to PIIV- and adjuvant-vaccinated mice with red indicating upregulated and blue indicating downregulated genes. **(F)** Protein-protein interaction network of top PIV-associated DEGs at 28 dpv generated using STRING analysis. **(G)** Bar graph showing the number of genes enriched in the top pathways associated with PIV at 28 dpv.

To investigate the molecular mechanisms underlying the differential protective immunity induced by PIV and PIIV vaccination, we compared the transcriptional profiles of splenocytes isolated from mice vaccinated with 10 µg PIV or 10 µg PIIV formulated with Alum, or Alum alone, at 7 and 28 dpv using RNA-seq analysis. Multidimensional scaling (MDS) was first used to visualize overall variation among samples in the RNA-seq dataset. In MDS plots, greater distances reflect higher transcriptional dissimilarity, while samples from similar treatment groups are expected to cluster together and separate from distinct groups. As shown in **Figure 5A**, RNA samples from PIV- and PIIV-vaccinated mice at 7 dpv clustered closely but away from naïve control (C7) and alum-immunized mice (A7), indicating highly similar transcriptional profiles in PIV and PIIV-vaccinated mice at this early time point. In contrast, at 28 dpv, splenocytes from PIIV- and Alum-vaccinated mice (PIIV28 and A28) clustered more closely with each other and were clearly separated from PIV-vaccinated samples (PIV28), indicating that PIV vaccination induced a distinct transcriptional signature compared to PIIV-vaccination at the later time point.

To assess data quality and model variance, the *voom* method was applied to estimate the mean–variance relationship of log-transformed counts and generate precision weights for downstream empirical Bayes analysis using the limma pipeline. Gene-wise means and variances are shown as black points with a LOWESS trend line (**Figure 5B**). The broad dispersion of points reflects substantial biological variation across PIV-, PIIV-, Alum-, and control-vaccinated groups. Differential gene expression analysis revealed distinct sets of upregulated (**Figure 5C**) and downregulated (**Figure 5D**) genes in splenocytes from PIV- and PIIV-vaccinated mice at 7 and 28 dpv, demonstrating that the two vaccines differentially modulate gene expression over time.

Consistent with flow cytometry based cellular findings (**Figure 4**), differentially expressed gene (DEG) analysis showed that genes associated with neutrophil-mediated responses were strongly upregulated in splenocytes from both PIV- and PIIV-vaccinated mice at 7 dpv (**Figure 5E**). By 28 dpv, however, neutrophil-related gene expression remained elevated only in splenocytes from PIV-vaccinated mice, whereas no significant differences were observed between PIIV- and Alum-vaccinated groups. These transcriptional patterns closely mirrored the flow cytometry results, indicating that PIV vaccination induces a sustained neutrophil response that is not maintained following PIIV vaccination.

Among the upregulated genes, early neutrophil activation markers *S100a8* and *S100a9*, which promote neutrophil aggregation and inflammatory responses, were elevated in splenocytes from PIV-vaccinated mice at both 7 and 28 dpv. In contrast, their expression in PIIV-vaccinated mice was increased at 7 dpv but returned to levels comparable to Alum-vaccinated controls by 28 dpv. Similarly, genes encoding chitinase-like proteins (*Chil1*, *Chil3*), lactoferrin (*Ltf*), the C-type lectin receptor *Clec4e* (Mincle), and cathelicidin (*Camp*) remained upregulated at 28 dpv exclusively in PIV-vaccinated mice suggesting sustained neutrophil effector functions.

Additional genes associated with neutrophil function and host defense, including *Csta*, *Stfa2*, *Stfa2l1*, *Olfm4*, *Olfml2b*, *Tcap*, *Retn*, *Prss34*, and *Lrg1*, were significantly upregulated in splenocytes from PIV-vaccinated mice at 28 dpv but were downregulated or unchanged in PIIV- and Alum-vaccinated mice. Notably, type I interferon–associated genes (*Ifitm1*, *Ifitm6*, *Ifitm7*) were strongly upregulated at 28 dpv in PIV-vaccinated splenocytes but not in PIIV-vaccinated mice. In contrast, chemokines (*Cxcl2*, *Ccl2*, *Ccl3*, *Ccl4*) were highly expressed in both vaccine groups at 7 dpv; by 28 dpv, their expression declined in PIV-vaccinated mice and was markedly reduced in PIIV-vaccinated mice, suggesting that while PIV sustains type I interferon–associated responses, it does not fully maintain neutrophil-associated chemokine expression at later time points. Collectively, these results demonstrate that PIV and PIIV differentially regulate neutrophil-associated gene expression during the later stages of vaccination, with PIV promoting a more persistent neutrophil activation program.

Protein–protein interaction analysis using STRING revealed that most PIV-specific upregulated genes form interconnected networks involved in host defense responses, particularly inflammatory signaling and granulocyte migration (**Figure 5F**). Reactome pathway analysis further showed selective enrichment of innate immune pathways in PIV-vaccinated splenocytes, including NOD1/2 signaling, interleukin-1 family signaling, Toll-like receptor signaling, neutrophil degranulation, and metal sequestration mediated by antimicrobial proteins (**Figure 5G**). Notably, several enriched protein domains—such as cystatin, cathelicidin, and chitinase families—are closely associated with antimicrobial effector functions. Together, these findings provide further evidence that PIV and PIIV differentially modulate neutrophil-associated signaling pathways, which may underlie their distinct protective efficacies against infection.

### Cell composition modeling identifies neutrophils as the key different responders between PIV- and PIIV- vaccinated mice

To complement the flow cytometry analysis, the computational deconvolution tool seq-ImmuCC was used to estimate the relative proportions of major immune cell types from the splenocyte RNA-seq data. This analysis included macrophages, mast cells, monocytes, neutrophils, eosinophils, dendritic cells, natural killer cells, B cells, CD8^+^ T cells, and CD4^+^ T cells. After processing and filtering the RNA-seq datasets, a reference signature matrix comprising 162 immune cell–specific genes was applied.

**Figure 6:**
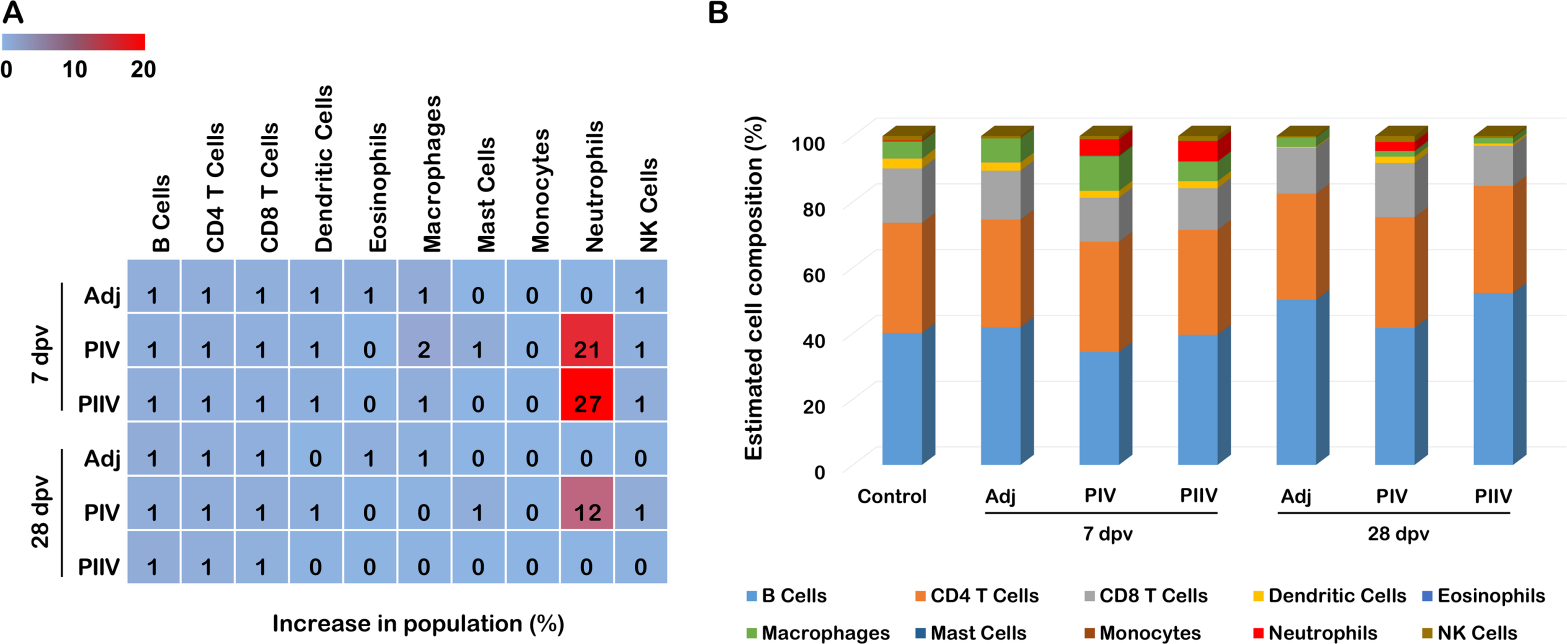
Computational deconvolution of splenic immune cell composition in PIV-, PIIV-, and adjuvant-vaccinated mice. **(A)** Heatmap showing fold differences in predicted immune cell populations at 7 and 28 dpv relative to naïve controls, with red indicating higher and purple indicating lower estimated abundance. **(B)** Stacked bar plot displaying the estimated percentage composition of each immune cell type, with corresponding color codes shown below.

As shown in **Figure 6A**, compared with Alum-vaccinated mice, neutrophil-associated gene signatures were increased in splenocytes from both PIV- and PIIV-vaccinated mice at 7 days post-vaccination (dpv). By 28 dpv, however, elevated neutrophil proportions were maintained only in splenocytes from PIV-vaccinated mice, whereas neutrophil levels in PIIV-vaccinated mice returned to those observed in Alum controls.

Analysis of overall immune cell composition (**Figure 6B**) revealed that variations observed in multiple immune cell populations at 7 days post-vaccination (dpv) in both PIV- and PIIV-vaccinated mice were largely driven by changes in neutrophil abundance, however, neutrophil abundance was observed only in PIV-vaccinated mice at 28 dpv. In contrast, B cells and CD4^+^ T cells consistently constituted the largest fractions of splenocytes across all experimental groups, irrespective of vaccination status, consistent with the expected cellular composition of the spleen and supporting the validity of the computational deconvolution approach. Taken together, these computational estimates were consistent with the flow cytometry findings and provide additional support for the conclusion that PIV and PIIV differ in their capacity to induce and sustain neutrophil responses, which may contribute to their distinct protective efficacies.

### Neutrophils contribute to PIV-induced protection against virulent C. burnetii NMI infection

**Figure 7:**
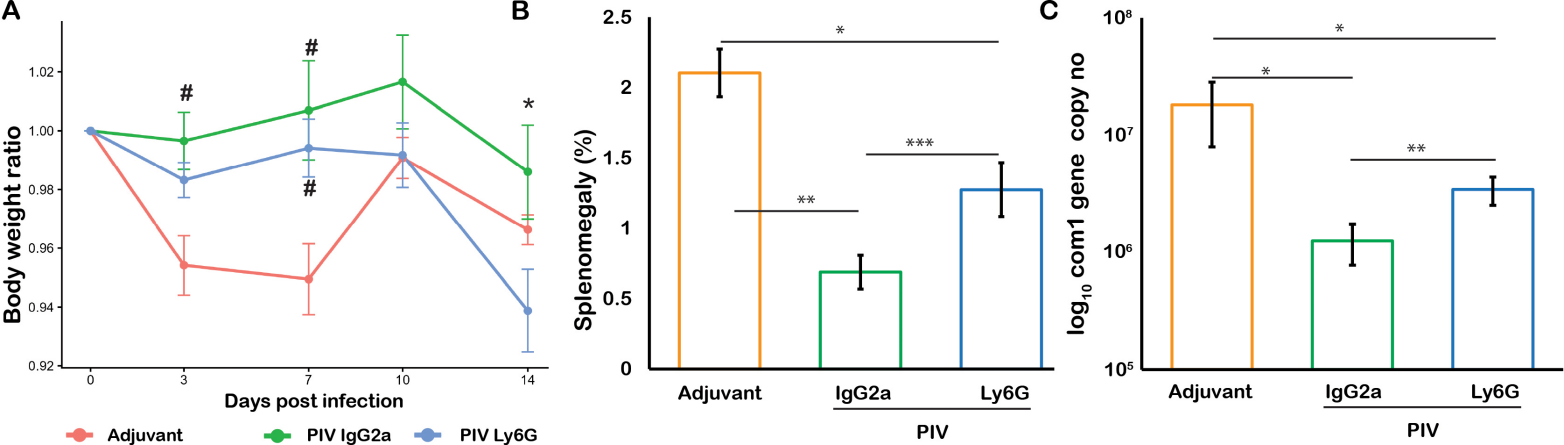
Effects of anti-Ly6G treatment-mediated depletion of neutrophils in PIV-vaccinated mice prior to NMI challenge. Mice vaccinated with PIV were treated with anti-Ly6G or isotype control antibody one day prior to NMI challenge; adjuvant-treated mice challenged with NMI served as controls. **(A)** Body weight ratio on days 3, 7, 10, and 14 post-challenge, shown relative to body weight on the day of challenge (Day 0). **(B)** Splenomegaly (spleen weight/body weight, %) measured at 14 dpi. **(C)** Bacterial burden in spleen at 14 dpi, quantified as relative *CbCom1* genomic copies normalized to mouse *tfrc* by qPCR. Statistical significance was determined using one-way ANOVA; ***p < 0.001, **p < 0.01 and *p < 0.05.

To assess the contribution of neutrophils to PIV-induced protective immunity, neutrophils were depleted in PIV-vaccinated mice prior to challenge with virulent *C. burnetii* NMI. As shown in **Figure 7A**, all PIV-vaccinated mice were largely protected from infection-induced transient body weight loss at 3 and 7 dpi, regardless of treatment with isotype control or neutrophil-targeting Ly6G antibody. By 14 dpi, however, Ly6G-treated mice exhibited a significant reduction in body weight compared with isotype-treated controls. Ly6G-treated PIV-vaccinated mice exhibited higher levels of splenomegaly (**Figure 7B**) and increased *C. burnetii com1* genomic copy numbers in the spleen (**Figure 7C**) compared with isotype-treated PIV-vaccinated mice. However, both Ly6G and isotype-treated PIV-vaccinated groups showed significantly lower splenomegaly and bacterial burden than Alum-vaccinated controls, indicating that PIV vaccination provides protection against infection. These results indicate that neutrophil depletion partially diminishes the protective efficacy of PIV vaccination, supporting a role for neutrophil-mediated effector functions in PIV-induced immunity against *C. burnetii* infection.

## Discussion

This study provides a detailed comparative analysis of the immune responses elicited by PIV and PIIV vaccines and identifies potential mechanisms underlying their differential protective efficacy against *Coxiella burnetii* NMI infection in mice. Consistent with previous studies (8, 21), formalin-killed NMI (PIV) provides robust protection against NMI infection, significantly reducing infection-induced splenomegaly and bacterial burden following both single and booster immunization schedules, whereas PIIV failed to provide similar protection regardless of the regimen (20, 25). These observations establish that PIV and PIIV differ fundamentally in their capacity to induce effective immunity.

PIV uniquely elicited early IgM and high, sustained IgG responses, suggesting a more effective antigenic profile. Although PIIV also induced antibody production against NMI antigens, the lack of protection indicates that the antigenic repertoire of PIIV is insufficient for protective immunity. This is consistent with previous reports that antibodies against O-antigen are critical for the protection against virulent *C. burnetii* (26).

Beyond antibody production, both PIV and PIIV induced significant splenomegaly and increased number of splenocytes at 3, 7 and 28 dpv suggesting that both vaccines induced significant immune response in spleen, consistent with previous findings (27). The spleen acts as a critical secondary lymphoid organ that filters blood-borne antigens, making it a primary site for initiating immune responses to systemic vaccines. It contains a high density of both lymphoid (T and B cells) and myeloid cells (neutrophils, macrophages, dendritic cells) that are activated by vaccinated antigens. Flow cytometry and seq-ImmuCC analyses on transcriptional data revealed robust neutrophil recruitment in spleen in both PIV- and PIIV-vaccinated mice at 7 dpv, however, only PIV sustained high neutrophil levels at 28 dpv, suggesting PIV-vaccine specific effects on neutrophils. Flow cytometric analysis of splenocytes from PIV-vaccinated mice at 28 dpv revealed a significant increase in CD11b^+^Ly6G^+^ and CD11b^+^CD125^+^ populations. Notably, the CD11b^+^CD125^+^ subset largely overlapped with Ly6G^+^ cells (**Supplementary Figure 4**), suggesting a sustained neutrophilic response, consistent with other findings (28). Further studies are needed to clarify the specific role of interleukin 5 receptor, CD125 expression in splenic immune cell populations. Transcriptomic and pathway analyses further revealed that genes associated with neutrophil degranulation, metal sequestration, and TLR-dependent innate activation remained highly expressed at 28 dpv only in PIV spleens, a pattern commonly detected in vaccinated spleens using bulk RNA-Seq, reflecting inflammation-associated innate immune activation (29). Neutrophil degranulation and other long-term functional changes in neutrophils have been demonstrated as critical mechanism for protective immunity in whole cell vaccines (30). These findings indicate that PIV uniquely maintains elevated neutrophil response long term. The sustained activation suggests that the O-antigen component of LPS in PIV may act as a critical agonist that prolongs neutrophil-driven innate signaling. Previous studies demonstrated that O-antigen from bacterial LPS is critical for chronic neutrophil activation (31, 32). Splenic neutrophils are crucial for polysaccharide-conjugate vaccine-induced protection, especially against bacterial infections, suggesting a critical role for O-antigen polysaccharide component of PIV in priming splenic neutrophils in conferring protection against virulent *C. burnetii* (33). This suggests that polysaccharides mimicking O-antigen can be used as an adjuvant to induce persistent neutrophil activation to increase the efficacy of other vaccines including peptide vaccines (34). Collectively, these data show that PIV induced persistent innate immune activation, and persistent neutrophils activation are critical for protection against virulent *C. burnetii*. Notably, the upregulation of neutrophil effector-associated molecules, including cathelicidin, cystatin, and chitinase family members, in the spleen at 28 dpv following PIV vaccination suggests enhanced innate immune activation and potential antimicrobial capacity. While these changes are consistent with innate effector responses (35), aligning with the reduced protection during neutrophil depletion, further mechanistic studies are required to define the activation kinetics and functional capacity of neutrophils, including TLR-activation, assessment of phagocytosis, reactive oxygen species (ROS) production, and neutrophil extracellular trap (NET) formation at 28 dpv, to establish the effector functions of these persistent splenic neutrophils and analyzing serum biochemical parameters might provide more insights on the toxicity induced by PIV at 28 dpv.

Overall, this study demonstrates that PIV vaccination induces sustained neutrophil recruitment and prolonged innate activation with enhanced effector-like functions, features absent in PIIV-vaccinated mice. Neutrophil depletion further reveals that PIV-induced neutrophils are required for efficient reduction of pathogen burden and inflammation during NMI challenge. These findings identify neutrophils as central cellular mediators of PIV-induced protection and outline a mechanistic framework involving persistent innate activation, antimicrobial gene networks, and cytokine modulation.

A key limitation of this study is that the bulk RNA-seq data were not sufficient to determine whether the Ly6G^+^ cells expanded after vaccination exhibit a specific phenotype. Moreover, the transcriptomic landscape was strongly neutrophil-skewed due to their dominant recruitment and activation, which may have masked transcriptional changes in low-abundance but biologically relevant populations such as eosinophils, previously demonstrated in PIV-mediated protection (20) and other splenic cell types whose roles remain undefined. More resolved approaches, such as transcriptomics on sorted cell populations and single-cell transcriptomics, will be needed to identify additional discrete cellular subtypes that contribute to the protective response. Although the persistent neutrophil abundance at 28 dpv and effector-like transcriptional phenotype were identified, it is important to explore mechanistic background of the effector functions.

## Data Availability

The datasets presented in this study can be found in online repositories. The names of the repository/repositories and accession number(s) can be found below: https://hgdownload.cse.ucsc.edu/goldenpath/mm10/bigZips/, GRCm38, Ensembl. Raw gene-level counts were normalized using HTSeq with 0.75 quartile normalization, log_2_-transformed, and are available in the Gene Expression Omnibus (GEO) under accession number GSE315405.
