# Supplementary material for: Systems biology approach unveils the cellular and molecular mechanisms of formalin-inactivated whole cell vaccine-induced protective immunity against Coxiella burnetii infection in mice
Source: Front Immunol. 2026 Jun 4;17:1809423. doi: 10.3389/fimmu.2026.1809423 (PMC13275218; doi:10.3389/fimmu.2026.1809423)

**Supplementary Figure 1:** Absolute count of different cell types in splenocytes.


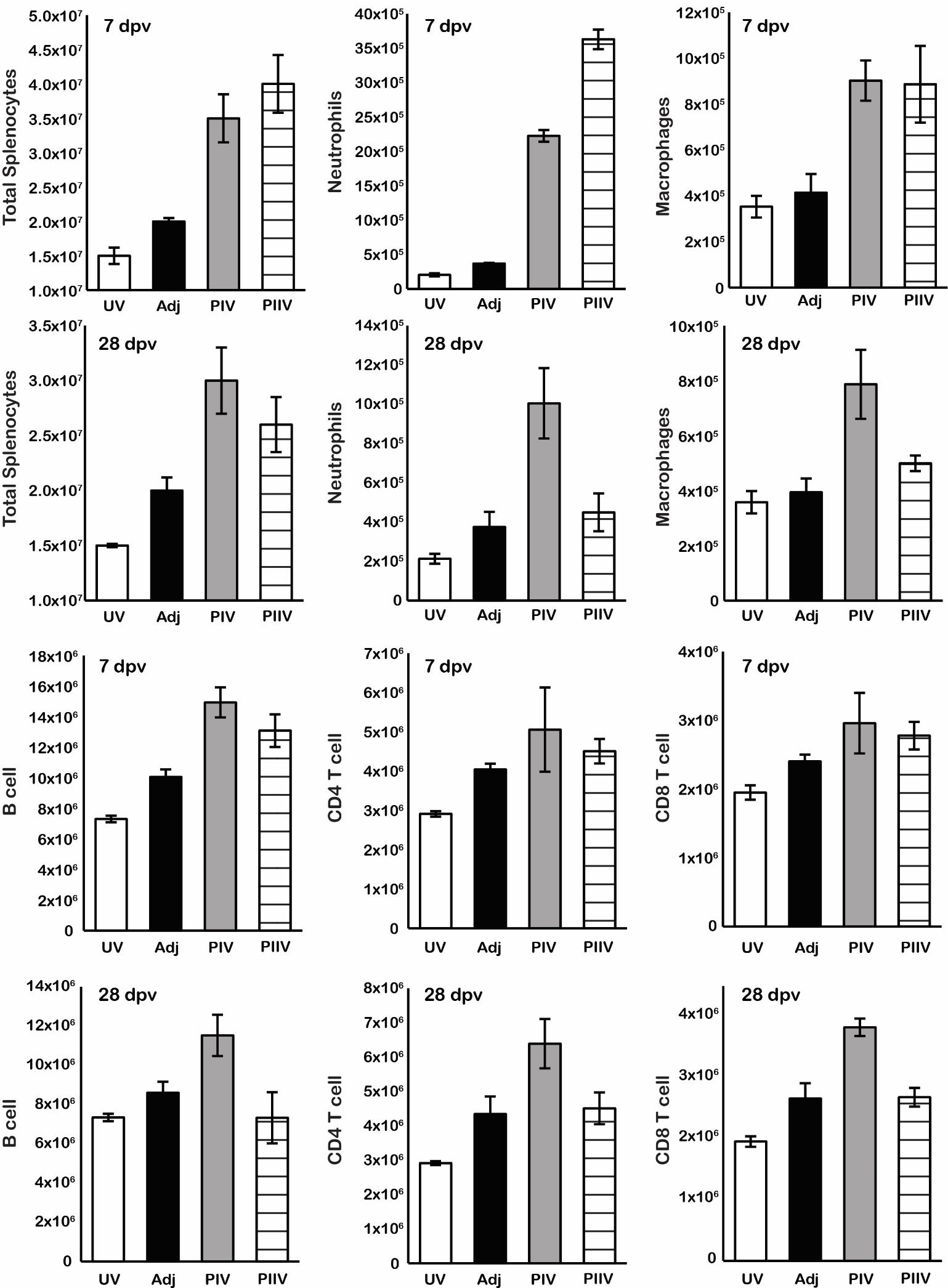


**Supplementary Figure 2**: Gating strategy of Panel 1: T cells


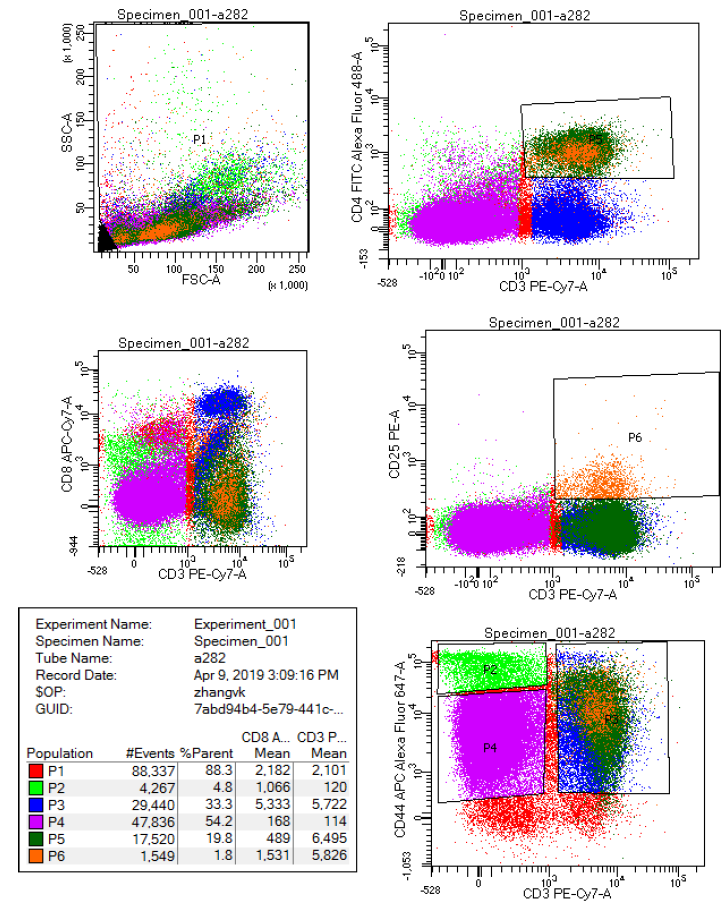


**Supplementary Figure 3**: Gating strategy of Panel 2: B cells


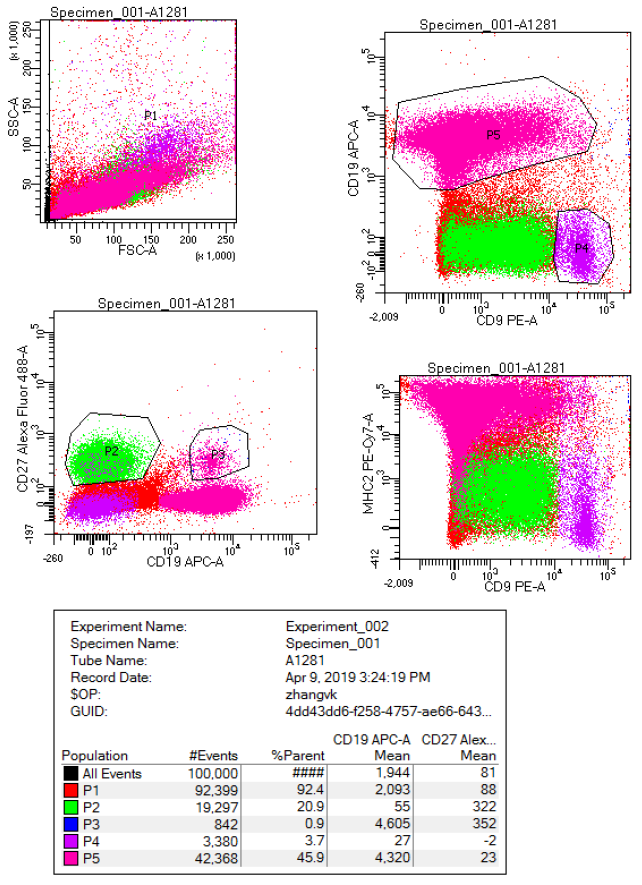


**Supplementary Figure 4**: Gating strategy of Panel 3: Myeloid cells


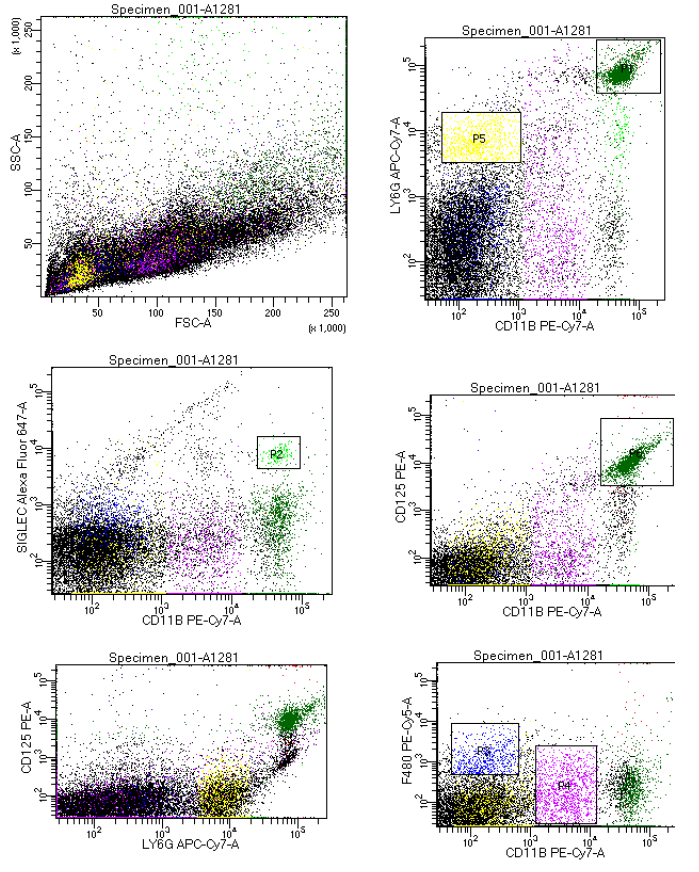

Supplement: Supplementary file 1 [file SupplementaryFile1.docx]
